# ET1 acts as a potential plasma biomarker and therapeutic target in deep venous thrombosis rat model

**DOI:** 10.1007/s11239-024-02981-4

**Published:** 2024-06-02

**Authors:** Zhanqi Wang, Zhangmin Wu, Zhongzhou Hu, Huanqin Zheng, Zhong Chen

**Affiliations:** Department of Vascular Surgery, Beijing Institute of Heart Lung and Blood Vessel Diseases, Beijing Anzhen Hospital of Capital Medical University, Beijing, 100029 China

**Keywords:** Deep venous thrombosis, Endothelin-1, Diagnostic marker, BQ123, The inferior vena cava stenosis

## Abstract

Deep venous thrombosis (DVT) is the third leading cause of death in cardiovascular disease, following heart attacks and strokes. Early diagnosis and intervention are crucial for effective DVT therapy. We aim to investigate whether endothelin-1 (ET-1) could serve as an early diagnostic marker or a potential therapeutic target in a DVT rat model. CCK8 assay, invasion assay, and flow cytometry were used to detect the proliferation, migration and apoptosis of HUVECs, respectively. Elisa assay was used to detect ET-1 and coagulation factor VII in cell supernatant and rat?s plasma. Western blot was used to detect antioxidant signaling protein. Inferior vena cava stenosis was used to construct the DVT rat model. Lentivirus mediated overexpression of ET-1 in HUVECs impaired the cell proliferation and migration, increased cell apoptosis, inhibited the antioxidant signaling pathway proteins expression (e.g., NQO1, GCLC, Nrf-2), and upregulated coagulation factor VII. Furthermore, overexpression of ET-1 further impaired antioxidant signaling pathway protein in response to H_2_O_2_ treatment. However, lentivirus mediated ET-1 knockdown and BQ123 (an ET-1 inhibitor), showed the opposite results with ET-1 overexpression. We then established a DVT rat model by inferior vena cava stenosis. The stenosis induced early expression of ET-1 and coagulation factor VII in plasma at day 1 and restore their level at day 10. BQ123 could downregulate the coagulation factor VII to ameliorate the stenosis effects. Our findings suggest that ET-1 might serve as an early diagnostic marker for DVT rat model and a potential therapeutic target for treating DVT.

**Supplementary Information:**

The online version contains supplementary material available at 10.1007/s11239-024-02981-4.

## Introduction

The deep venous thrombosis (DVT), which refers to a blood clot that forms within the deep veins, usually in the leg, is a common multifactorial disease. It significantly affects the patient’s quality of life in both acute and chronic phases and can be life-threatening [1]. The incidence rate is as high as 1.2% and continues to increase each year [2]. Currently, plasma D-dimer is the only molecular marker used for diagnosing the disease, but it only helps exclude the diagnosis in some patients [3]. Due to the insidious symptoms in the early stages, the diagnosis is often missed. Therefore, the development of effective and early diagnostic markers is crucial for diagnosing and preventing DVT.

A recent study discovered that the continuous release of vasoconstrictor substances could facilitate clot formation, making it one of the primary factors in the development of deep venous thrombosis [4]. Additionally, our previous findings demonstrated that the expression of endothelin-1 (ET-1) in plasma increased when vascular endothelial cells were injured by stimuli, leading to higher expression of endothelial adhesion molecules and damage to the antioxidant pathway [5]. ET-1 is an important vasoconstrictor secreted by endothelial cells and is involved in arteriosclerosis and vascular remodeling. Endothelin, a peptide consisting of three types of endothelins (ET-1, ET-2, and ET-3), activates the G protein-coupled receptors ETA and ETB [6], with ET-3 having a slightly weaker effect in activating ETA subtypes [7]. ET-1 is the most abundant endothelium isoenzyme in the human cardiovascular system, distributed in endothelial cells of the lungs, kidneys, and colon, as well as macrophages of peripheral glial cells and monocytes of the central nervous system. However, the most extensively studied are the monolayers of endothelial cells in the cardiovascular system, which are covered by the vascular wall and have powerful endocrine glandular functions [8]. This peptide, encoded by a gene that exists only in vertebrates, activates the ligand receptor signaling pathway in vertebrates. ET-1 is one of the potent vasoconstrictors. As a potential vasoconstrictor factor secreted by endothelial cells, it is continuously activated in the human cardiovascular system, and binds with receptors to exert its vasoconstrictive role. It regulates the normal contractile function of blood vessels by maintaining the contractile state of smooth muscle cells [9]. Studies applying low expression of ET-1 through sub-effective alleles and high expression of ET-1 through super-effective alleles have confirmed that overexpression of ET-1 and ET-2 in many tissues could accelerate the processes of inflammation and fibrosis, especially in the cardiovascular system [10]. Therefore, even minor changes in ET-1 levels can lead to vascular dysfunction. Based on these studies, we hypothesize that ET-1 may serve as an early diagnostic marker and a potential therapeutic target for DVT.

The purpose of this study was to evaluate whether ET-1 could indeed function as an early diagnostic marker and a potential therapeutic target for DVT. In vitro study demonstrated that lentivirus mediated overexpression of ET-1 in human umbilical vein endothelial cells (HUVECs) impaired the cell proliferation and migration, increased cell apoptosis, inhibited the antioxidant signaling pathway proteins expression (e.g., NQO1, GCLC, Nrf-2), and upregulated the secreted level of coagulation factor VII. To simulate the conditions of DVT, we induced inferior vena cava stenosis in rats, which resulted in increased levels of ET-1 and coagulation factor VII expression in plasma at an early stage of thrombosis. Furthermore, the administration of BQ123 was found to mitigate these effects in the DVT rat model. To the best of our knowledge, this is the first study reporting the potential of ET-1 as an early diagnostic marker and a potential therapeutic target in DVT rat model.

## Methods

### Cell culture

HUVECs (iCell-h110) were obtained from iCell Bioscience Inc, Shanghai. HUVECs were cultured in culture medium (iCell-h110-001b). Cells were passaged every 4 days with 0.25% trypsin. Medium was changed daily. Cells were routinely checked for normal morphology, the bacterial or mycoplasma contamination. Passages 3–10 of HUVECs were used for assay.

### Lentivirus plasmid construction and package

The END1 sequence and its shRNA sequence were respectively cloned into pSLenti-CMV-END1-3xFLAG-PGK-Puro-WPRE and pCLenti-U6-shRNA1-CMV-Puro-WPRE. The interference sequences are listed in Supplementary Table 1. The virus packaging was performed using the packaging kit from SBI (Mountain View, CA, USA) and according to the manufacturer’s instructions. The virus particles were harvested and concentrated using PEG-it virus precipitation solution (SBI). Virus titer was determined in 293TN cells using the Global UltraRapid Lentiviral Titer Kit (SBI), according to the manufacturer’s instructions. HUVECs were infected with lenti-END1, lenti-sh-END1 and their Lenti-control, respectively, at a different multiplicity of infection in the presence of Polybrene (5 µg/mL; Sigma).

### CCK8 assay

Collect cultured HUVECs and adjust cell concentration to 5 × 10^4^ cells/mL, then 100 µL/well of cell suspension from different groups was inoculated in a 96-well plate. Pre-incubate the plate in a humidified incubator at 0 h, 24 h, 72 h and 120 h. In drug treating group, 10 µM BQ123 was added. Add 10 µl of the Cell Counting Kit-8 solution to each well of the plate. Incubate the plate for 2 h in the incubator. Measure the absorbance at 450 nm using a microplate reader.

### Cell invasion assay

Human umbilical vein endothelial cells (HUVECs) were harvested and washed with 3 ml PBS. The cells were digested and collected with 0.25% trypsin, centrifuged at 1000 rpm for 5 min, and rinsed twice with PBS. The cells were re-suspended in serum-free medium and counted by cell counting plate. The cells were diluted to 3 × 10^5^/ml in serum-free medium. The Matrigel was melted at 4 °C one day earlier, and the Transwell Chamber, 24-well culture plate and gun tip were precooled at -20 °C overnight. Matrigel was diluted to the final concentration of 1 mg/ml in serum-free medium, and operated on ice. 800 µl 10% FB medium was precooled at 4 °C in 24-well plates and placed in a transwell chamber. 100 ΜL Matrigel with a final concentration of 1 mg/ml was added vertically at the bottom of the upper chamber of the Transwell Chamber. Matrigel was incubated at 37 °C for 4–5 h to make it dry and gelatinous. After Matrigel was dried and gelatinous, 200 µl cell suspensions of each group were placed in the Transwell Chamber and cultured at 37 °C and 5% CO_2_ for 72 h in the chamber. Transwell was removed, the chamber was carefully washed with PBS, and the cells were fixed with 70% ice-ethanol solution for 1 h. The cells were stained with 0.5% crystal violet solution, placed at room temperature for 20 min, washed with PBS, and wiped with a clean cotton ball to clean the non-migrated cells on one side of the upper chamber and the other side.

### Cell apoptosis

The HUVECs from different groups were collected, the trypsin was digested with EDTA-free medium and collected at room temperature at 1000 rpm for 5 min, collect cells. Cells were resuspended once with precooled 1xPBS (4 °C), centrifuged at 1000 rpm for 5 min, and washed. Add 300 ΜL of 1x Binding Buffer suspension cells. Annexin V-FITC was mixed with 5 µl of Annexin V-FITC and incubated at room temperature for 15 min. After adding 10 µl PI staining, the cells were incubated at room temperature for 10 min in the dark. Flow cytometry was used to test and analyze results.

### RNA extraction and quantitative real-time polymerase chain reaction

Total RNA was extracted from the cultured cells using Trizol reagent (Invitrogen, Carlsbad, CA, USA) following the manufacturer’s protocol. The extracted RNA was quantified using a NanoDrop 2000 spectrophotometer (Thermo Scientific, Bremen, Germany). cDNAs were synthesized from the mRNA using TIANScript RT (Tiangen Biotech Co., Ltd, Beijing, China), with oligo (dT) primers used for reverse transcription. For qRT-PCR, the 7500 System (ABI) was utilized. SYBR Premix (F-415XL, Thermo) was used for the qRT-PCR reactions. The specific forward and reverse primers employed for reverse transcription and qRT-PCR are listed in Supplementary Table 2. All reactions were performed in triplicate. The relative mRNA expression levels were determined using the 2^(-ΔΔCt) method and normalized to the expression levels of the endogenous reference gene, glyceraldehyde-3-phosphate dehydrogenase (GAPDH).

### Western blot analysis

For Western blot analysis, total protein was extracted from the cultured cells using lysis buffer. 40 µg of the protein sample were separated by SDS polyacrylamide gel electrophoresis and subsequently transferred onto nitrocellulose membranes. The membranes were then incubated overnight at 4 °C with primary antibodies targeting specific proteins, including NQO1 (1:1000, ab80588, abcam), GCLC (1:1000, ab20777, abcam), Nrf2 (1:1000, ab62352, abcam). To ensure accuracy and consistency, the anti-GAPDH antibody (1:5000, 60004-1-Ig, Proteintech) was included as a loading control in each individual experiment. After three washes, the membranes were incubated with horseradish peroxidase-conjugated secondary antibodies (1:7000, ZB2305, Zhongshanjinqiao Biotechnology Co., Ltd) for 1 h at room temperature. The antigen-antibody complexes were detected using enhanced chemiluminescence, and the resulting images were analyzed using a Chemiscope 5300 Pro (CLINX).

### H&E staining

The inferior vena cava thrombosis tissues of rat were isolated and fixed in 4% PFA solution for two days, sequentially dehydrated with 70%, 95% and 100% ethanol, and defatted with xylene for 2 h before being embedded in paraffin. The 10-µm-thick section was cut and subjected to hematoxylin and eosin staining (H&E staining). The slices were observed under optical microscopy.

### Enzyme-linked immunosorbent assay (ELISA)

Cell culture supernatant and plasma concentrations of ET-1 (D711033) and coagulation factor VII (MM-50735H2) were quantified using commercially available enzyme-linked immunosorbent assay (ELISA) kits. The detection range of ET-1 kit was from 5 pg/mL to 160 pg/mL, and the lowest detection concentration was less than 1.0 pg/mL. The detection range of coagulation factor VII kit was from 1.5625 ng/mL to 50 ng/mL, and the lowest detection concentration was less than 0.1 ng/mL. The measurements were performed in accordance with the manufacturers’ instructions, and each sample was assessed in triplicate to ensure accuracy. The absorbances at 450 nm were recorded, and the mean concentrations were calculated based on these measurements.

### Rat model of deep venous thrombosis (DVT) using inferior vena cava stenosis

Under anesthesia, the rats’ intestine and mesentery were repositioned to the right side, followed by the surgical opening of the retroperitoneal cavity. Subsequently, the junction of the left renal vein and the inferior vena cava (IVC), along with the segment of the IVC located 1 cm below it, were meticulously separated. A number 0 silk ligature was then carefully passed around the left renal vein, securing both ends of the ligature through the right side of the abdominal wall, thereby facilitating extrusion from the abdominal cavity. Similarly, a ligature was applied to the common iliac vein. The bowel and its associated mesentery were subsequently reintegrated into the abdominal cavity, ensuring appropriate adjustment of the ligature position to prevent bowel compression. The two ligature lines were individually secured onto the abdominal wall, and 2 ml of normal saline was administered into the abdominal cavity to conclude the abdominal closure. Attention was paid to the tension of the ligature; inadequate tightness may result in insufficient blood flow blockade and modeling failure, whereas excessive tightness may induce abdominal wall retraction and intestinal compression, leading to adverse outcomes. The adequacy of ligature tension was calibrated to permit passage of a standard 2B pencil. Postoperatively, the animals were maintained under appropriate warmth and provided unrestricted access to food and water. After excising the IVC, thrombi can be measured and then snap frozen or formalin-fixed for further analysis. For BQ-123 group (3 mg/kg), it was delivered into DVT rat model by tail vein injection.

### Statistical analysis

The data are presented as the mean ± standard deviation (SD) from three independent experiments. We used an unpaired t-test to compare two groups and one-way ANOVA to compare more than two groups. A sample size of 3 was selected so that at a significance level of 0.05 there at least 95% chance of detecting two SD’s difference in outcome between the groups. All data were processed using GraphPad Prism 8 (GraphPad Software, Inc., La Jolla, CA). All analyses were conducted using SAS software (version 9.3, 2002–2012; SAS Institute Inc., Cary, NC, USA). Statistical significance was defined as *P* < 0.05 for all comparisons.

## Results

### ET-1 affects HUVECs’ proliferation, invasion and apoptosis

**Fig. 1:**
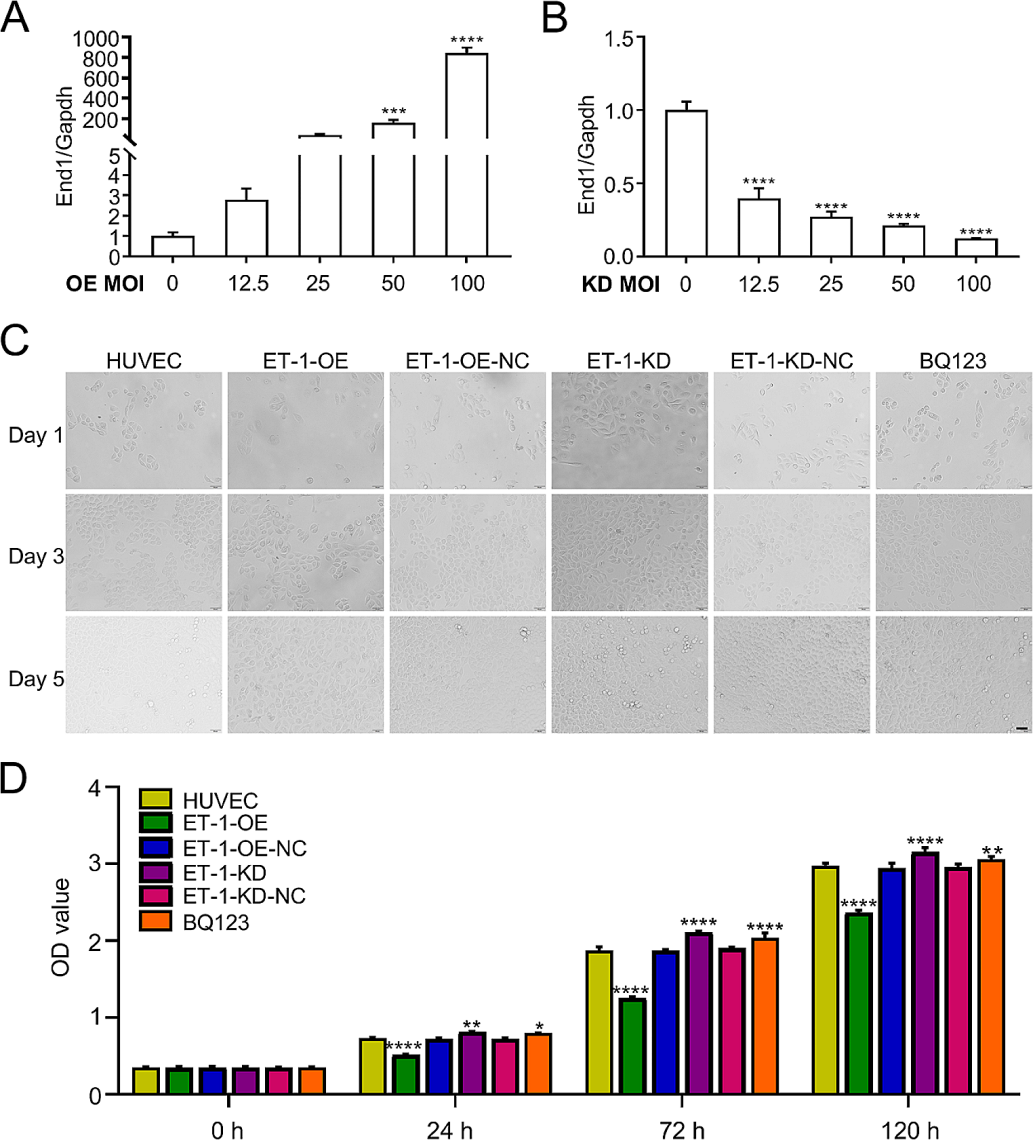
The effect of ET-1 on HUVECs proliferation. (**A, B**) qRT-PCR validation of ET-1 expression in HUVEC by lentivirus mediated ET-1 overexpression (**A**) and lentivirus mediated shRNA-knockdown (**B**). (**C, D**) Cell morphologies at day 1, 3 and 5, and CCK8 assay at 0 h, 24 h, 72 h and 120 h, showing ET-1 overexpression significantly inhibited HUVECs proliferation, while ET-1 knockdown and BQ123 (an ET-1 inhibitor) could rescue cell proliferation compared to HUVECs control. Scale bars, 50 μm

**Fig. 2:**
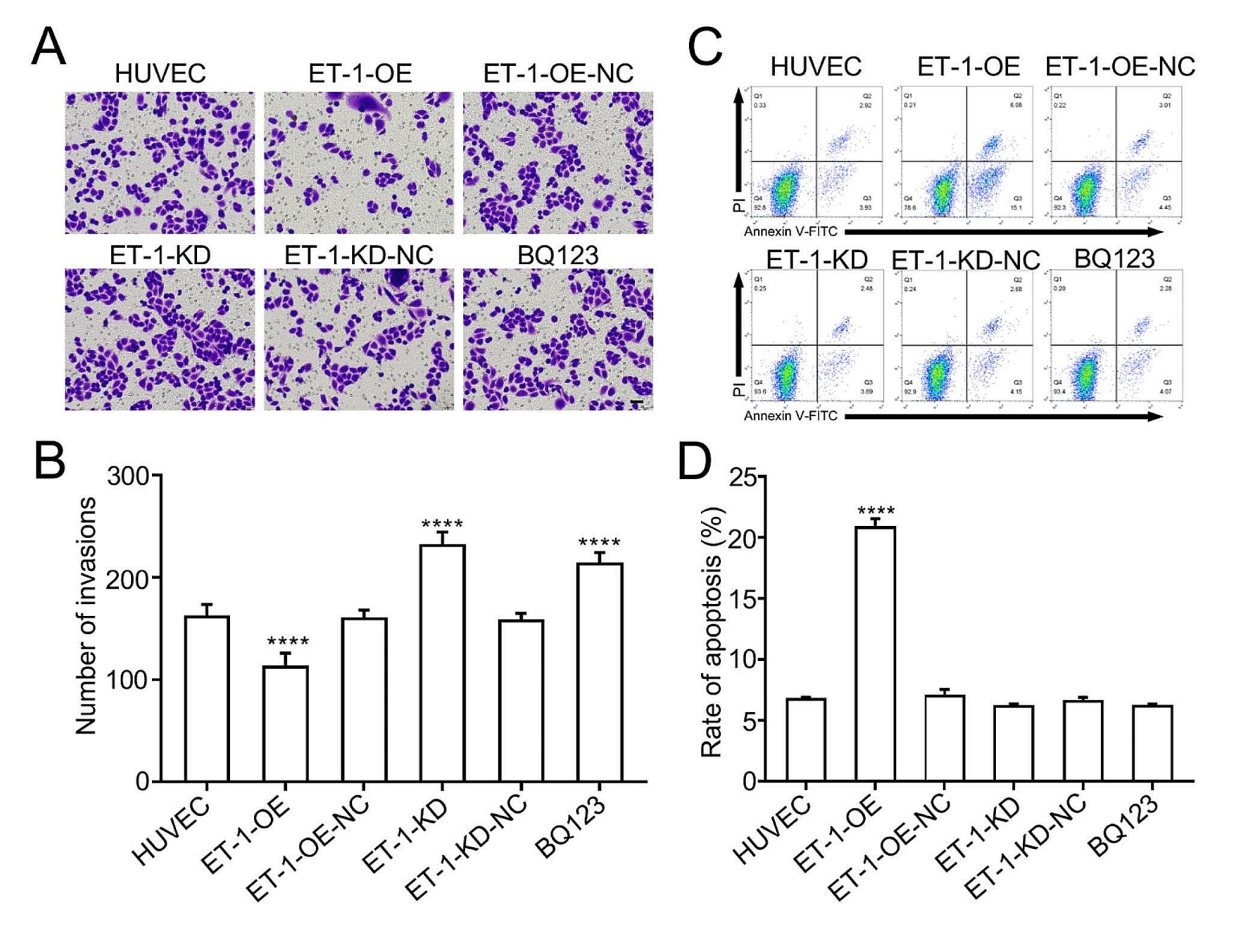
The effect of ET-1 on HUVECs migration and apoptosis. (**A, B**) Cell invasion assay and statistical analysis showing ET-1 overexpression significantly decreased cell migration, and ET-1 knockdown and BQ123 increased cell migration. Scale bars, 50 μm. (**C, D**) Flow cytometry and statistical analysis showing ET-1 overexpression significantly increased the apoptosis of HUVEC, while ET-1 knockdown and BQ123 administration did not affect HUVEC’s apoptosis

Our previous study demonstrated a significant increase in the expression level of ET-1, as determined by enzyme-linked immunosorbent assay analysis of blood samples from both a rat model of smoke-induced vascular injury and human smokers [5]. Additionally, it has been reported that damage to endothelial cells contributes to the development of deep vein thrombosis (DVT) [11]. Therefore, we hypothesize that ET-1 may play a crucial role in the progression of DVT. To investigate the underlying molecular mechanism of DVT, our study initially focuses on elucidating the functional role of ET-1 in human umbilical vein endothelial cells (HUVECs). The experiment involved four groups: HUVECs with lentivirus mediated ET-1 overexpression, HUVECs with lentivirus mediated ET-1 knockdown, HUVECs treated with BQ123 (an ET-1 inhibitor), and HUVECs as a negative control. Firstly, we validated the expression of ET-1 in the intracellular compartments of all four groups using qRT-PCR analysis. Our results confirmed an gradually increase in ET-1 levels in HUVECs with different MOI of lentivirus mediated ET-1 overexpression (Fig. 1A), and a gradually decrease in ET-1 expression in HUVECs with different MOI of lentivirus mediated ET-1 knockdown (Fig. 1B) compared to HUVECs. Regarding functional assays, our findings indicated that ET-1 overexpression inhibited HUVECs proliferation (Fig. 1C, D) and invasion (Fig. 2A, B), while enhanced HUVECs apoptosis (Fig. 2C, D). Conversely, knockdown of ET-1 expression resulted in increasing HUVECs proliferation and invasion, along with a decrease in HUVECs apoptosis (Figs. 1C and D and 2A-D). These results strongly suggest that ET-1 significantly influences the functions of HUVECs.

### ET-1 negatively regulates Nrf-2 anti-oxidant pathway

**Fig. 3:**
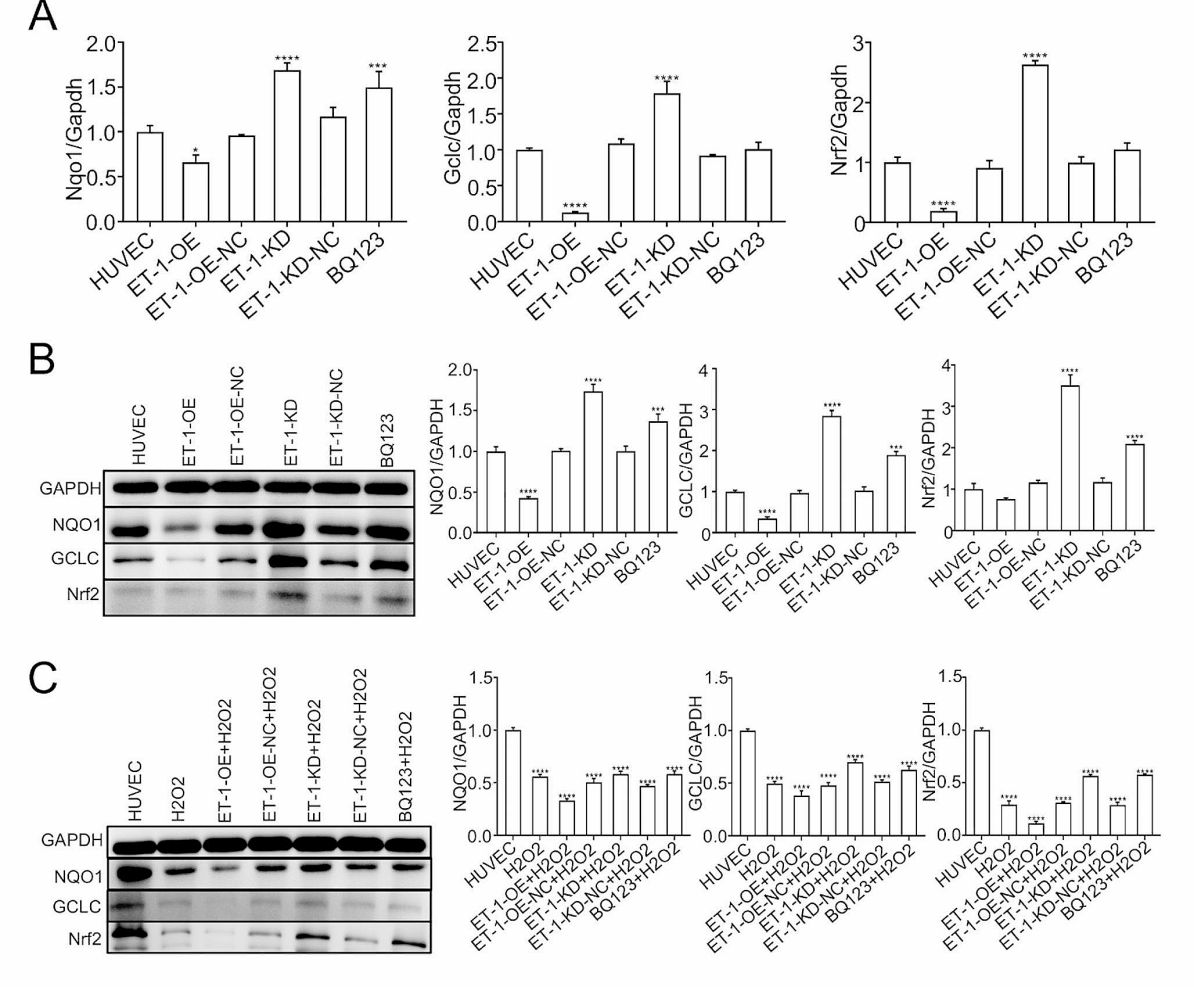
The effect of ET-1 on antioxidant signaling pathway proteins. (**A**) qRT-PCR analysis of mRNA level of the antioxidant signaling protein, Nqo1, Gclc and Nrf2. (**B**) Western blot and statistical analysis showing ET-1 overexpression significantly decreased NQO1, GCLC and Nrf2 protein level, while ET-1 knockdown and BQ123 could upregulate NQO1, GCLC and Nrf2 protein level. (**C**) To further investigate the effect of ET-1 on antioxidant signaling pathway protein, after constructing the stable HUVEC with overexpressed ET-1 or knockdown ET-1, and simultaneously treating with 1% H2O2, followed by detecting NQO1, GCLC and Nrf2 protein level

**Fig. 4:**
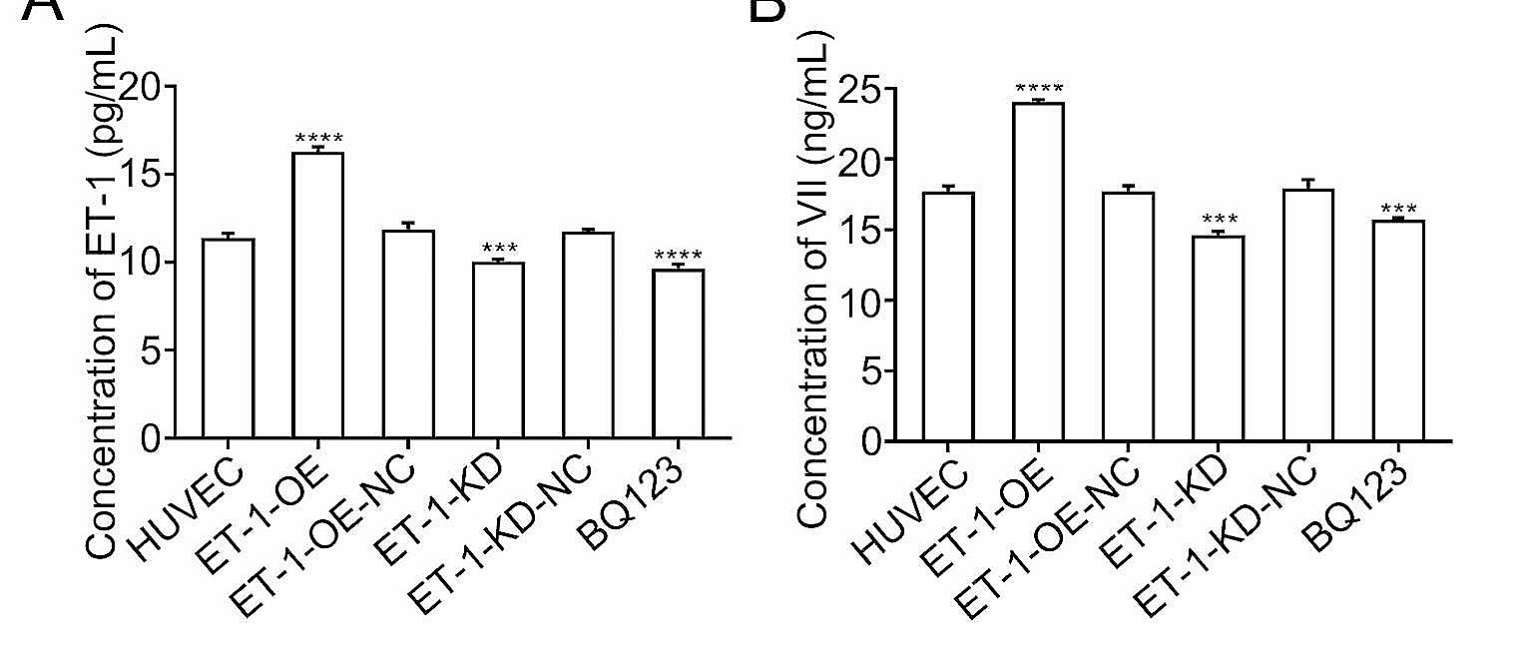
The effect of ET-1 on the secretion of ET-1 and coagulation factors VII. (**A, B**) Elisa assay showing ET-1 overexpression significantly increased the secreted level of ET-1 (**A**), and coagulation factor VII (**B**) in cell culture supernatant, while ET-1 knockdown and BQ123 significantly inhibited those two factors secretion

Nrf2, a transcription factor characterized by a basic leucine zipper motif, exhibits heightened sensitivity to oxidative stress and assumes a protective role in various organs during states of oxidative stress [12, 13]. Numerous studies have demonstrated that activating Nrf2 through pharmacological or genetic means significantly mitigates cellular damage resulting from oxidative stress and serves as a preventive measure against the development and progression of stress-induced diseases [14]. In our previous investigation, we provided evidence that melatonin alleviates smoke-induced vascular injury by modulating the Nrf-2 antioxidant pathway [5]. Consequently, to explore the potential effect of ET-1 on the Nrf-2 antioxidant pathway in human umbilical vein endothelial cells (HUVECs), we assessed the mRNA levels of Nrf-2, NQO1, and GCLC, which are downstream targets of Nrf2 involved in reducing oxidative damage during the protective process [15]. Our findings revealed that overexpressing ET-1 significantly suppressed the mRNA and protein levels of Nrf-2, NQO1, and GCLC, as determined by qRT-PCR (Fig. 3A) and western blot analysis (Fig. 3B), whereas ET-1 knockdown resulted in an upregulation of these three proteins, as did treatment with BQ123 (Fig. 3A, B). Additionally, to further investigate the relationship between ET-1 overexpression and the Nrf-2 antioxidant pathway, we exposed all four groups to 1% H_2_O_2_. Notably, H_2_O_2_ could further decrease antioxidant pathway protein expression in HUVECs with overexpressed ET-1. Conversely, HUVECs with ET-1 knockdown and BQ123 administration could significantly rescue the expression of Nrf-2, NQO1, and GCLC (Fig. 3C). Additionally, we found that overexpression of ET-1 could significantly increase the secretion of ET-1 and coagulation factor VII in the cell culture supernatant by Elisa assay (Fig. 4A, B). Overall, these results collectively indicate that ET-1 exerts a negative regulatory effect on the antioxidant pathway in HUVECs.

### ET-1 acts as an early plasma diagnostic marker

**Fig. 5:**
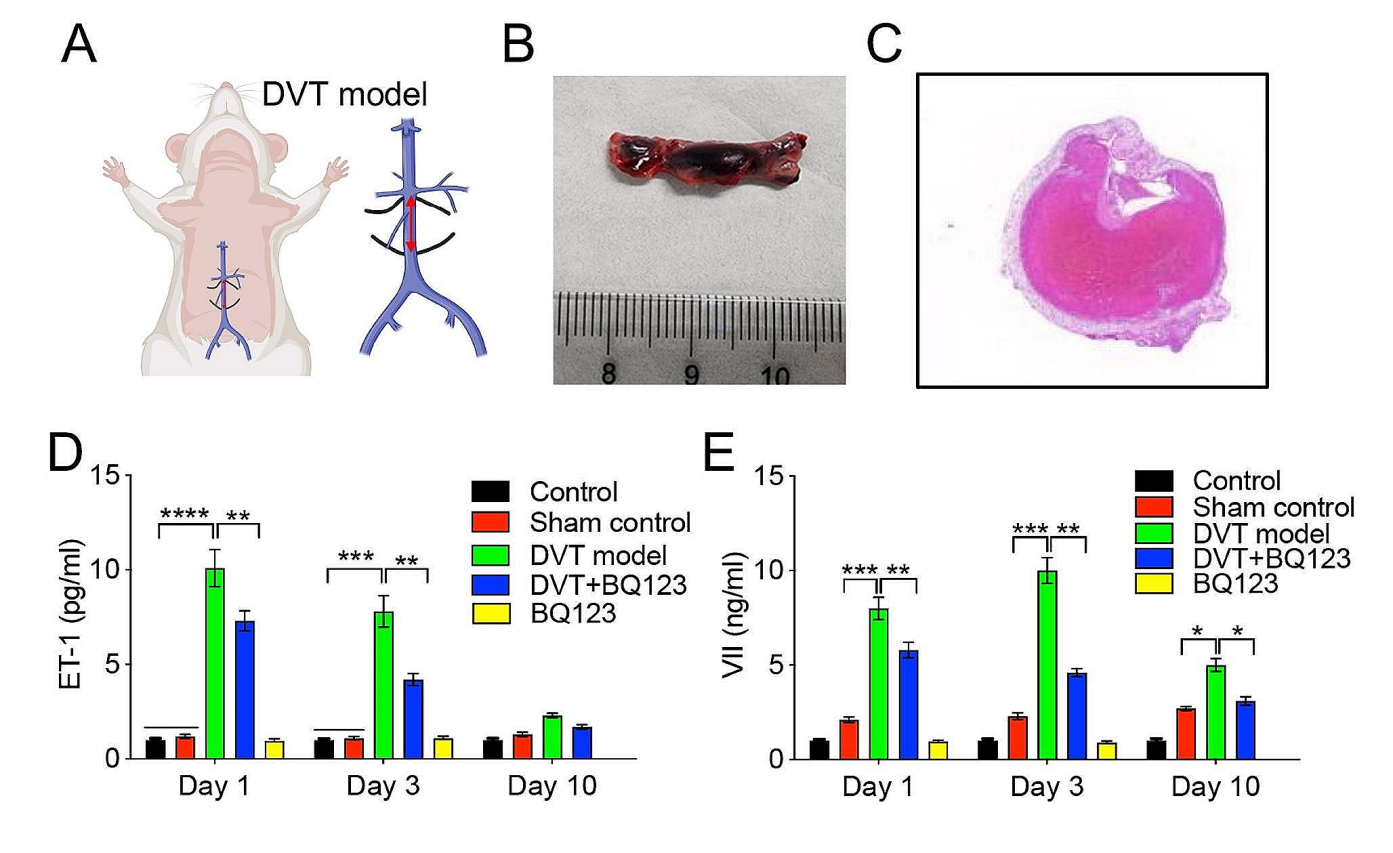
The ET-1 might acts as an early diagnostic marker in plasma of DVT rat model. (**A**) Schematic illustration of construction of DVT rat model through inferior vena cava stenosis. (**B**) The cut off thrombosis (∼2 cm) showing the success of constructing DVT rat model. (**C**) H&E staining showing the thrombosis formation blocked the blood vessel. (**D, E**) Elisa assay showing ET-1 and coagulation factor VII was significantly increased in plasma at early day 1, while BQ123 administration (3 mg/kg) could significantly decrease both of two factors to ameliorate DVT syndrome

In order to investigate the potential utility of ET-1 as an early plasma diagnostic marker, we established a rat model of deep venous thrombosis (DVT) using inferior vena cava stenosis (Fig. 5A) and confirmed the success of the procedure through H&E staining (Fig. 5B, C). The experiment encompassed four groups: normal control, sham control, DVT model, and BQ123 administration (3 mg/kg). Plasma samples were collected from all four groups at postoperative days 1, 3 and 10. Elisa assay revealed a significant upregulation of ET-1 and coagulation factor VII levels as early as day 1 in DVT rat model group, while the elevated level of ET-1 and coagulation factor VII gradually decrease to close to the normal level over time (Fig. 5D, E). But, BQ123 administration could significantly inhibited ET-1 and coagulation factor VII and lasted to day 10 (Fig. 5D, E). In addition, we added only BQ-123 group for treating normal rat on day 1 and day 3 compared with normal control to evaluate its adverse effect. We found that BQ-123 did not affect ET-1 and coagulation factor VII levels (Fig. 5D, E). These findings indicate that ET-1 holds promise as an early diagnostic marker and a potential therapeutic target for deep venous thrombosis.

## Discussion

This study aimed to investigate the molecular mechanisms underlying the involvement of ET-1 in deep venous thrombosis (DVT). DVT is a significant contributor to cardiovascular disease-related mortality, ranking third among the leading causes [16]. The occurrence of DVT is closely associated with imbalances in the coagulation and fibrinolysis systems, endothelial damage, and sluggish blood flow [11]. The initiation of hemostasis involves the disruption of the venous wall and endothelial injury, and in the context of DVT, this process can occur even in the absence of wall damage [17, 18]. Nevertheless, the precise molecular mechanisms underlying DVT remain unclear. In our previous study, we observed a significant increase in the expression level of ET-1 in blood samples from both a rat model of smoke-induced vascular injury and human smokers [5]. As the most potent vasoconstrictor secreted by endothelial cells, ET-1 is normally present in low concentrations in the bloodstream and responds to the stimulus of cigarette smoke (CS). Recent research has established a correlation between blood ET-1 concentration and atherosclerosis and vascular remodeling [19]. Investigations utilizing alleles with suboptimal ET-1 expression and alleles with hyperactive ET-1 expression have confirmed that heightened expression of ET-1 and ET-2 in various tissues can exacerbate inflammation and fibrosis processes, particularly in the cardiovascular system [10]. Consequently, even minor alterations in ET-1 levels can lead to vascular dysfunction. Building upon these studies, we propose the hypothesis that ET-1 may serve as an early diagnostic marker and a potential therapeutic target for DVT. Moreover, our intracellular findings provide further confirmation of the impact of ET-1 on the functional aspects of human umbilical vein endothelial cells (HUVECs).

Subsequently, in order to further elucidate the molecular mechanism underlying the impact of ET-1 on HUVEC functions, we directed our focus towards investigating the Nrf-2 antioxidant signaling pathway. Our previous investigation demonstrated a significant downregulation of Nrf2 expression levels, along with its downstream genes, following exposure to smoking, which was subsequently reversed upon administration of melatonin in both smoke-exposed rats and human smokers. Nrf2, recognized as an antioxidative transcription factor, is deemed crucial for conferring resistance against atherosclerosis and exhibits in vitro binding to antioxidant response element (ARE) sequences [20]. The Nrf2/Keap1-ARE signaling pathway represents the most potent endogenous antioxidative pathway and exerts remarkable antioxidant effects [21]. Multiple studies have provided evidence that Nrf2 activation through pharmacological or genetic means substantially diminishes cellular damage induced by oxidative stress and effectively prevents the development and exacerbation of stress-induced diseases [14]. Notably, Nrf2 knockout mice display inherent susceptibility to oxidative stress-induced diseases [22]. In the present study, we further demonstrated that overexpressed ET-1 significantly decreased the protein levels of Nrf-2, NQO1, and GCLC, indicating that ET-1 exerts a negative regulatory effect on the Nrf-2 antioxidant pathway in HUVECs.

Subsequent to this, in order to evaluate the potential of ET-1 as an early diagnostic marker, we established a rat model of DVT by inducing inferior vena cava stenosis. Plasma levels of ET-1 were assessed at various time points, including early day 1, day 3 and day 10. The findings revealed a substantial increase in ET-1 and coagulation factor VII levels as early as day 1, while the elevated level of ET-1 and coagulation factor VII gradually decrease to close to the normal level over time. However, it is important to note that further verification of these results in human subjects is warranted.

## Conclusion

In conclusion, the current study demonstrated that ET-1 exerts influence over HUVECs by modulating their proliferation, invasion, and apoptosis, while also inhibiting the Nrf-2 anti-oxidant signaling pathway. Additionally, in the rat model of DVT, a substantial elevation in plasma ET-1 levels was observed as early as day 1. Based on our findings, we propose that ET-1 holds promise as both an early diagnostic marker and a potential therapeutic target in DVT rat model.

### Electronic supplementary material

Below is the link to the electronic supplementary material.

Supplementary Material 1

## References

[1] Kucher N (2013) Deep-vein thrombosis of the Upper extremities. 364:861–869. 10.3238/arztebl.2017.0244 10.1056/NEJMcp1008740 21366477

[2] Nguyen E, Caranfa JT, Lyman GH et al (2018) Clinical prediction rules for mortality in patients with pulmonary embolism and cancer to guide outpatient management: a meta-analysis. J Thromb Haemost 16:279–292. 10.1111/jth.13921 29215781 10.1111/jth.13921

[3] Keller K, Beule J, Balzer JO, Dippold W (2018) D-Dimer and thrombus burden in acute pulmonary embolism. Am J Emerg Med 36:1613–1618. 10.1016/j.ajem.2018.01.048 29371044 10.1016/j.ajem.2018.01.048

[4] Wakefield TW, Myers DD, Henke PK (2008) Mechanisms of venous thrombosis and resolution. Arterioscler Thromb Vasclar Biol 28:387–391. 10.1161/ATVBAHA.108.162289 10.1161/ATVBAHA.108.162289 18296594

[5] Wang Z, Ni L, Wang J et al (2016) The protective effect of melatonin on smoke-induced vascular injury in rats and humans: a randomized controlled trial. J Pineal Res 60:217–227. 10.1111/jpi.12305 26681403 10.1111/jpi.12305

[6] Speed JS, D’Angelo G, Wach PA et al (2015) High salt diet increases the pressor response to stress in female, but not male ETB-receptor-deficient rats. Physiol Rep 3:1–7. 10.14814/phy2.12326 10.14814/phy2.12326 PMC4393160 25802361

[7] Barrera-Chimal J, Prince S, Fadel F et al (2016) Sulfenic acid modification of Endothelin B receptor is responsible for the benefit of a nonsteroidal mineralocorticoid receptor antagonist in renal ischemia. J Am Chem Soc 27:398–404. 10.1681/ASN.2014121216 10.1681/ASN.2014121216 PMC4731121 26361797

[8] Semaan W, Desbiens L, Houde M et al (2015) Chymase inhibitor-sensitive synthesis of endothelin-1 (1–31) by recombinant mouse mast cell protease 4 and human chymase. Biochem Pharmacol 94:91–100. 10.1016/j.bcp.2015.02.001 25667044 10.1016/j.bcp.2015.02.001

[9] Hathaway CK, Grant R, Hagaman JR et al (2015) Endothelin-1 critically influences cardiac function via superoxide-MMP9 cascade. Proc Natl Acad Sci U S A 112:5141–5146. 10.1073/pnas.1504557112 25848038 10.1073/pnas.1504557112 PMC4413291

[10] Maguire JJ, Davenport AP (2015) Endothelin receptors and their antagonists. Semin Nephrol 35:125–136. 10.1016/j.semnephrol.2015.02.002 25966344 10.1016/j.semnephrol.2015.02.002 PMC4437774

[11] Chougule A, Rajpal S, Ahluwalia J et al (2016) Coagulation factor VIII, IX and XI levels in north Indian patients with venous thromboembolism: first study from India. Blood Coagul Fibrinolysis 27:58–63. 10.1097/MBC.0000000000000390 26340461 10.1097/MBC.0000000000000390

[12] Vriend J, Reiter RJ (2015) The Keap1-Nrf2-antioxidant response element pathway: a review of its regulation by melatonin and the proteasome. Mol Cell Endocrinol 401:213–220. 10.1016/j.mce.2014.12.013 25528518 10.1016/j.mce.2014.12.013

[13] Kensler TW, Wakabayashi N, Biswal S (2007) Cell survival responses to environmental stresses via the Keap1-Nrf2-ARE pathway. Annu Rev Pharmacol Toxicol 47:89–116. 10.1146/annurev.pharmtox.46.120604.141046 16968214 10.1146/annurev.pharmtox.46.120604.141046

[14] Kim Y-S, Zhuang H, Koehler RC, Doré S (2005) Distinct protective mechanisms of HO-1 and HO-2 against hydroperoxide-induced cytotoxicity. Free Radic Biol Med 38:85–92. 10.1016/j.freeradbiomed.2004.09.031 15589375 10.1016/j.freeradbiomed.2004.09.031

[15] Satsu H, Chidachi E, Hiura Y et al (2012) Induction of NAD(P)H: Quinone oxidoreductase 1 expression by cysteine via Nrf2 activation in human intestinal epithelial LS180 cells. Amino Acids 43:1547–1555. 10.1007/s00726-012-1230-1 22302369 10.1007/s00726-012-1230-1

[16] Wolberg AS, Rosendaal FR, Weitz JI et al (2015) Venous thrombosis. Nat Rev Dis Prim 1:15006. 10.1038/nrdp.2015.6 27189130 10.1038/nrdp.2015.6

[17] Luxembourg B, Pavlova A, Geisen C et al (2014) Impact of the type of SERPINC1 mutation and subtype of antithrombin deficiency on the thrombotic phenotype in hereditary antithrombin deficiency. Thromb Haemost 111:249–257. 10.1160/TH13-05-0402 24196373 10.1160/TH13-05-0402

[18] Ochodnicky P, Henning RH, van Dokkum RPE, de Zeeuw D (2006) Microalbuminuria and endothelial dysfunction: emerging targets for primary prevention of end-organ damage. J Cardiovasc Pharmacol Suppl 2:S151–162. 10.1097/00005344-200606001-00009 10.1097/00005344-200606001-00009 16794452

[19] Schiffrin EL (2002) Beyond blood pressure: the endothelium and atherosclerosis progression. Am J Hypertens 15. 10.1016/s0895-7061(02)03006-6. :115S-122S 10.1016/s0895-7061(02)03006-6 12383592

[20] Howden R (2013) Nrf2 and cardiovascular defense. Oxid Med Cell Longev 2013. 10.1155/2013/104308 10.1155/2013/104308 PMC3649703 23691261

[21] Lee JM, Johnson JA (2004) An important role of Nrf2-ARE pathway in the cellular defense mechanism. J Biochem Mol Biol 37:139–143. 10.5483/bmbrep.2004.37.2.139 15469687 10.5483/bmbrep.2004.37.2.139

[22] Meakin PJ, Chowdhry S, Sharma RS et al (2014) Susceptibility of Nrf2-Null mice to Steatohepatitis and Cirrhosis upon Consumption of a high-Fat Diet is Associated with oxidative stress, perturbation of the unfolded protein response, and disturbance in the expression of metabolic enzymes but not with I. Mol Cell Biol 34:3305–3320. 10.1128/mcb.00677-14 24958099 10.1128/mcb.00677-14 PMC4135558

