# Supplementary material for: ET1 acts as a potential plasma biomarker and therapeutic target in deep venous thrombosis rat model
Source: J Thromb Thrombolysis. 2024 Jun 2;57(6):1067–75. doi: 10.1007/s11239-024-02981-4 (PMC11315785; doi:10.1007/s11239-024-02981-4)
Supplement: Supplementary file 1 — Supplementary Material 1 [file 11239_2024_2981_MOESM1_ESM.docx]

**Supplementary Table 1.**

| **shRNA** | **Sequence** |
| --- | --- |
| shRNA-1 | GCAGTTAGTGAGAGGAAGAAA |
| shRNA-2 | CCATGAGAAACAGCGTCAAAT |
| shRNA-3 | GCTCGTCCCTGATGGATAAAG |
| shRNA-Control | TTCTCCGAACGTGTCACGT |

**Supplementary Table 2. Primers use in this study.**

| **Name** | **Forward 5’-3’** | **Reverse 5’-3’** |
| --- | --- | --- |
| END1 | AACCCACTCCCAGTCCACCCT | CCAAGTCCATACGGAACAACG |
| NQO1 | AGACCTTGTGATATTCCAGTTCCCCCT | GGCAGCGTAAGTGTAAGCAAACTCTCC |
| GCLC | TGAAGCAATAAACAAGCAC | TTGGACAGATAGTAGCCAA |
| Nrf2 | AGACAAACATTCAAGCCGCT | AGACAAACATTCAAGCCGCT |
| GAPDH | GGAGCGAGATCCCTCCAAAAT | GGCTGTTGTCATACTTCTCATGG |
